# Sense of Coherence, Health, Well-Being, and Work Satisfaction before and after Implementing Activity-Based Workplaces

**DOI:** 10.3390/ijerph17145250

**Published:** 2020-07-21

**Authors:** Katarina Wijk, Eva L. Bergsten, David M. Hallman

**Affiliations:** 1 Centre for Research and Development, Region Gavleborg/Uppsala University, 801 87 Gavle, Sweden; 2 Faculty of Health and Occupational Studies, Department of Occupational Health Sciences and Psychology, University of Gavle, 801 76 Gävle, Sweden; eva.bergsten@hig.se (E.L.B.); david.hallman@hig.se (D.M.H.); 3 Department of Public Health and Caring Sciences, Uppsala University, 751 23 Uppsala, Sweden

**Keywords:** activity-based workplaces, implementation: well-being, sense of coherence, work satisfaction, health

## Abstract

Activity-based workplaces (ABWs) are implemented with possible implications for health, well-being, and work satisfaction in the workplace. Drawing on the theoretical framework, i.e., sense of coherence (SOC), the aim was to investigate how indicators pf SOC—meaningfulness, manageability and comprehensibility—are associated with, or function as barriers or facilitators for, health, well-being and work satisfaction during relocation to an ABW. We followed the implementation of ABWs at the Swedish Transport Administration (2018–2019). Questionnaires were administered before (*n* = 536), 3 months (*n* = 409) and 9 months (*n* = 373) after relocation. Focus group interviews (15) were conducted before and after. Data were analyzed using repeated measures ANOVA and content analysis. Relocation to an ABW was associated with a reduced work satisfaction (physical *p* < 0.001; psychosocial *p* < 0.001), and minor changes in health and occupational well-being during relocation (*p* > 0.001). The reduction in work satisfaction was smaller among employees with high meaningfulness in the relocation process (*p* < 0.001). All SOC indicators were positively associated with overall health, well-being and work satisfaction (*p* < 0.001). Interviews suggested that meaningfulness was facilitated by participation in the presented activities and that communication before relocation was crucial. The results indicate that organizations implementing ABWs should promote perceived meaningfulness in the process to mitigate possible declines in satisfaction with the physical and psychosocial work environment.

## 1. Introduction

Today’s technology enables flexibility in terms of where, when and how we can work, and there has been a need to adapt workspaces to these new ways of working [1]. Activity-based workplaces (ABWs) support various characteristics of today’s office work and might reduce costs through a more efficient use of office spaces [2]. ABWs are intended to provide office spaces that support different work characteristics such as silent, creative, and interactive work [3].

ABWs are typically divided into different zones, such as quiet zones for concentrated work and active zones for collaboration. Some of the characteristics for an ABW include unassigned and shared desks, with the possibility to change places in the office many times per day if desired, and when tasks shift. An ABW preferably has office rules and a clean desk policy, meaning that personal devices are removed when leaving the desk and then stored in lookers and toolboxes. 

Employees generally have a need to experience a sense of coherence and to understand why a change is made to be able to handle the change and feel that the change has meaning [4]. The salutogenic concept “Sense of coherence” (SOC) is a theoretical framework that explains how people manage stressful situations to maintain their health and well-being. SOC consists of three components: comprehensibility, manageability and meaningfulness [5]. Comprehensibility concerns feelings of how things make sense in terms of their coherence, structure and clarity [6]. Manageability is about the extent to which we feel that resources are at our disposal to meet the demands we are exposed to [6]. Meaningfulness refers to emphasizing the changes with engagement, i.e., view them as a challenge rather than a burden that should be avoided [6]. These three components of SOC act as personal resources that may protect individuals from stress and reduce health risks, as supported by empirical studies [7,8,9]. Thus, people with a higher SOC may be more likely to adapt to changes in the environment, and thereby be more resistant to declines in health, well-being and satisfaction at work [9]. Moreover, it has been shown that providing employees with a resourceful working environment helps to build their SOC and thereby increase work engagement [4]. With that starting point, one can assume that the perceptions of SOC during relocation to an ABW contribute to beneficial outcomes after relocation. Thus, the sense of coherence theory (SOC), developed by Antonovsky, was used as a conceptual framework for this study in order to study relocation.

This study followed the implementation of an ABW. A successful implementation generally requires certain prerequisites: it should include a planning phase and an idea of assumed change [10]. ABWs are implemented in many organizations, with implications for employees’ perceived physical and psychosocial working conditions [11,12]. Personal preferences seem to affect the use of workstations [13]. This highlights the need to further explore the perceptions of implementation in an ABW in varied groups. Previous research on the effects of ABWs has, to some extent, focused on the health, well-being and satisfaction with the office environment with results suggesting both positive and negative effects of an ABW [3,12,13,14,15]. In a systematic review by Engelen et al. [12], it was found that activity-based working had potentially positive effects on physical and psychosocial working conditions. However, evidence on the effects of ABWs on health and well-being were equivocal [12]. One possible reason for the conflicting results can be the differences in study designs. In addition, studies rarely consider the implementation process and its importance for a successful relocation [16]. A few studies found that process factors, e.g., information, had an impact on satisfaction with the office environment [17,18,19]. However, there are large differences in employee satisfaction with ABWs between cases [17]. This indicates the need to identify underlying factors that facilitate beneficial changes in perceptions during relocation to an ABW. Working conditions, such as working in cell offices versus working in an ABW, might lead to work-related consequences in terms of well-being, satisfaction, motivation and performance on the individual, team and organizational level where work tasks affect the outcome [11]. This indicates that the office environment matters, and one can assume that a relocation contributes to changes in those factors.

Engagement among employees prior to relocation may influence the way ABWs are perceived and utilized [20]. As an example of this, a previous intervention showed that the physical work environment and organizational culture affect behavior at work [21]. This also raises questions: for example, if other factors, such as attitudes towards relocation, affect one’s well-being in the workplace and health. Still, there is more to learn about in terms of how factors such as meaningfulness, manageability and comprehensibility during relocation impact health, well-being and work satisfaction [12]. 

Studies indicate that perceived performance and employee satisfaction with the physical environment might increase after relocation to an ABW [18]. There are also previous studies showing that employee empowerment relates to performance and environmental satisfaction [15]. Additionally, innovative offices, such as open-plan, flexible workplaces and paperless offices, had no or limited effects on work-related fatigue, health, and productivity, although they did have positive effects on general health [9]. For this reason, it is important to further study changes in health, well-being and work satisfaction both during implementation and after relocation to an ABW. 

There is a lack of research regarding the implementation process and underlying factors related to the health, well-being and satisfaction during the implementation of ABWs [11]. Research on the implementation process of ABWs is insufficient and there is a lack of concrete knowledge on whether meaningfulness, comprehensibility and manageability are factors that affect perceptions prior to relocation to an ABW and if they are associated with health, well-being and work satisfaction before and after relocation. Drawing on the SOC framework and previous research, we therefore addressed the employee perceptions of meaningfulness, comprehensibility and manageability concerning the implementation of ABWs, perception of facilitating factors and barriers, and the relationship with changes in health, well-being and work satisfaction after relocation. We expected that indicators for SOC would be of importance during the relocation process to an ABW, and buffer the negative effects of relocation on these outcomes. 

The aims of this study were:

(i) to investigate the extent to which indicators for SOC, health, well-being, and work satisfaction change during relocation to an ABW;
(ii) to shed light on employee perceptions of facilitating factors and barriers related to SOC during implementation;
(iii) to investigate the extent to which indicators for SOC during the process (i.e., before relocation to an ABW) are associated with changes in health, well-being and work satisfaction during relocation among office employees.

## 2. Materials and Methods

### 2.1. Design

This intervention study uses a prospective design to follow the implementation of ABWs [22]. Qualitative and quantitative data were collected in 2018 and 2019 using questionnaires administered before relocation, 3 months and 9 months after relocation. Focus group interviews were conducted with employees, a working group, safety delegates and managers before and after the relocation. All participants signed an informed consent form prior to participation. The study was approved by the Regional Ethical Review Board in Uppsala, Sweden (Ref. No. 2015/118).

### 2.2. The Intervention: Relocation to Activity-Based Workplaces (ABW)

The intervention was designed and implemented by the organization, without any involvement from the research group. The research group had previously been engaged in an evaluation of the relocation to ABWs at other geographical office sites in this organization (2014–2017) [23]. Thus, there was an established collaboration with the team responsible for initiating the current implementation at the agency. Researchers had solely the role of investigators and were commissioned to explore the implementation process and evaluate the effects on health, well-being and work satisfaction. The main incentive for implementing ABWs was to promote a stimulating, modern and digitalized work environment to increase collaboration and interaction in the workplace. Estimations at the agency in the present study indicated an office occupancy of around 30–40% before relocation. It was also assumed that costs would be reduced by using the offices more efficiently. 

Relocation was conducted during the summer vacation in August 2018. No other major structural changes or interventions were planned or implemented in the organization during the relocation. A project leader was tasked with overseeing the process of preparing the premises, including the office design and furniture. A working group was appointed to facilitate the process of relocation by preparing employees for an activity-based working method by organizing activities, providing information and managing questions from employees regarding the relocation. The working group was made up of representatives from the different departments, plus a safety delegate. 

Intervention Activities before Relocation to an ABW:

- Ergonomic seminars about the ABW design, activity-based work and how to eliminate risk factors for musculoskeletal injuries;
- Management information concerning the implementation of an ABW (why and how it will be achieved);
- Workshops in the management of knowledge and tools for utilizing ABWs;
- Inspiration seminars—aiming to inspire an activity-based approach to working.

### 2.3. Study Population

This study was conducted on 775 employees at one regional office site of a large governmental agency in Sweden. The average age was 44.9 years, 47% were woman (53% men), and 13% held a managerial position. Before the relocation, 32% worked in private offices, 11% in shared rooms (2–3 persons), 41% in an open-plan office (4–24 persons) and 16% in undefined places. The criteria for exclusion were employees on sick leave, parental leave or reporting career changes or retirement in advance. Employees at the agency that did not relocate or had received prioritized seats were also excluded. For the focus groups, the project manager and the working group members asked employees from different departments for their participation in the focus group interviews. If they agreed to participate, researchers sent them an invitation with the details for participation and a time for the interview.

**Figure 1:**
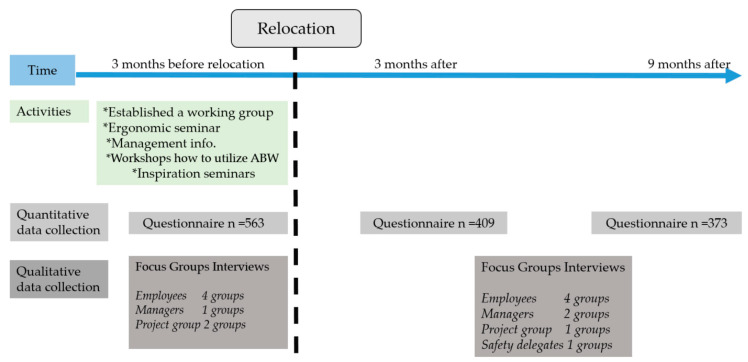
Timeline for implementation and data collection (2018–2019).

The response rate was 73% (*n* = 563) before relocation, 53% (*n* = 409) 3 months after, and 48% (*n* = 373) 9 months after. Two-hundred and forty-six persons answered the questionnaire on all three occasions (Figure 1). In total, 15 focus group interviews were conducted, seven before relocation and eight after relocation. In each group, an average of six to eight persons participated.

### 2.4. Data Collection

#### 2.4.1. Quantitative Data 

A questionnaire was sent out before and after relocation to measure the indicators for SOC regarding the relocation, health, well-being in the workplace and satisfaction with the physical and psychosocial work environment. Indicators for SOC regarding the relocation were measured using customized questions. Meaningfulness in terms of the relocation was measured using a question assessing the extent to which it was perceived that an ABW could contribute to the agency’s vision to improve productivity through stimulating, modern and digital work environment. Manageability for the implementation of an ABW was measured using a question about the support received from managers and co-workers to work and adjust to the new ways of working in ABWs [24,25]. Comprehensibility was measured using a question assessing the extent to which the respondents comprehended what it means to work in an activity-based way. A scale from not at all (1) to a very high extent (6), was used to gauge meaningfulness, manageability and comprehensibility. Self-assessed general health was measured by asking the question “In general, how would you like to say that your health is?” using a five point scale from 1 (excellent) to 5 (poor) [26]. For well-being, the question was “Here are some faces that express different degrees of well-being. Which face best expresses how you experienced your well-being in the workplace over the past four weeks?” (scale 1 (☺) to 7 (☹) [23]. Physical and psychosocial work satisfaction were also measured through the question “Regarding your work how satisfied are you in general with your physical work environment/psychosocial work environment?”. The scale ranged from 1 (very satisfied) to 5 (very unsatisfied) [27].

#### 2.4.2. Qualitative Data 

An interview guide was used together with questions addressing the implementation process of ABWs. The respondents were asked to relay what they thought constituted barriers or facilitation factors in the intervention process and their role in the process. They reflected on issues that might contribute to the meaningfulness, comprehensibility, and manageability in the transition process. The interviews lasted on average 60 min and were recorded electronically.

Eight of the focus groups interviews were conducted with workers from mixed groups of workers from different departments. The departments in question were planning, investments, central management, support, maintenance, purchase, logistics and communication. The working group appointed to oversee the process of implementation were interviewed three times. Separate interviews with the safety delegates were conducted. Three focus group interviews were conducted with managers. The mix of the groups was considered to provide an insight into the attitudes, opinions and reflections on the implementation and facilitate group interactions during the interviews.

Therefore, we wanted the groups to be homogenous in terms of participants’ potential assignments during the implementation, as in the example of the working group, safety delegates and managers. All groups were represented by different ages, genders and the extent to which the participants knew each other as colleagues from the same or different departments.

### 2.5. Analysis

A repeated measures ANOVA was used to determine the changes in indicators for sense of coherence, health, well-being and work satisfaction during the relocation to an ABW, using time (baseline, 3 months, 9 months) as a within-subject factor and health, well-being and work satisfaction (physical and psychosocial) as dependent variables. Participants responding to all three measurements were included in the study in order to investigate the changes during the relocation among employees. Respondents did not differ from non-responders in the studied factors. A mixed ANOVA was used to evaluate the effects of meaningfulness, comprehensibility and manageability on health, well-being and work satisfaction during the relocation to an ABW, using time as a within-subject factor and each measure of SOC as a between-subject factor (i.e., in separate models for meaningfulness, comprehensibility and manageability). In this analysis, we divided meaningfulness, comprehensibility, and manageability into three groups using tertiles (i.e., low, medium and high). The analysis scale was reversed so that higher numbers indicated positive outcomes. All analyses were conducted in SPSS, version 24.0 (IBM, Armonk, NY, USA). The level of significance was set at *p* < 0.05. Effect sizes were evaluated using partial eta square.

The interviews were transcribed, resulting in about 300 pages in total. All interviews were read repeatedly. The analysis was based on the methodology for content analysis [28,29], meaning that the analysis was focused on extrapolating various aspects of content from the transcribed records. The three components of SOC (i.e., manageability, comprehensibility, and meaningfulness) were used as a conceptual framework when analyzing the facilitating factors and barriers during the implementation of ABWs. In the first step, perceptions of the implementation process were highlighted, then divided into facilitating factors and barriers as they relate to the implementation of ABWs. The three components of SOC were then used to create categories. Two individuals categorized the interviews separately. This resulted in six possible contents: facilitating factors regarding manageability, comprehensibility, and meaningfulness and then barriers for these same concepts. Each category had subcategories that were based on excerpts from the interviews. 

## 3. Results

### 3.1. Changes in Indicators for SOC (Meaningfulness, Manageability, and Comprehensiveness), Health, Well-Being, and Work Satisfaction during Relocation to an ABW

**Figure 2:**
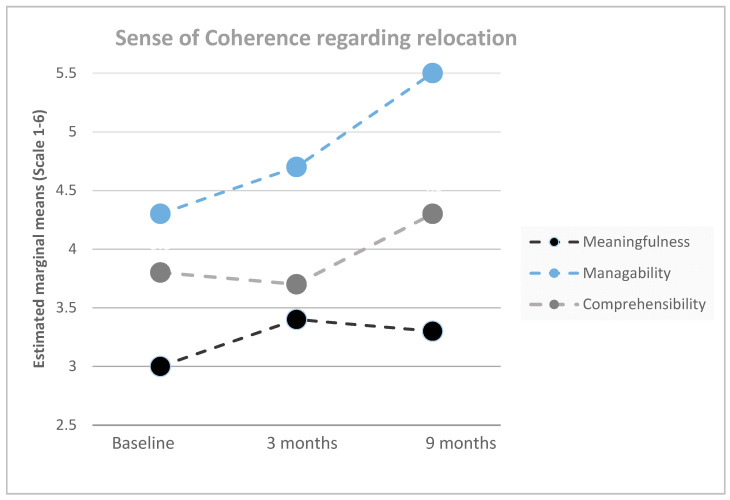
Estimated means (repeated measures ANOVA) of manageability, comprehensibility, and meaningfulness (scale 1–6) regarding the relocation to activity-based workplaces at baseline, 3 months, and 9 months after relocation (*n* = 246).

Indicators for SOC (i.e., manageability, comprehensibility, and meaningfulness) regarding the implementation of ABWs showed significant effects over time and increases after relocation (RM-ANOVA, all *p* < 0.001, see Table 1 and Figure 2). 

### 3.2. Changes in Health, Well-Being, and Work Satisfaction during Relocation 

**Figure 3:**
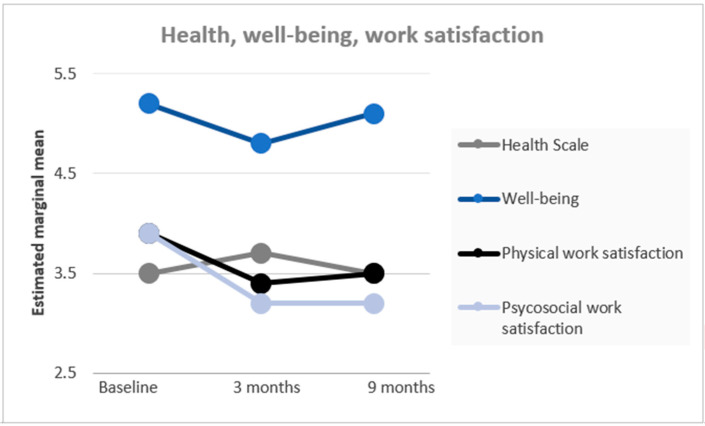
Estimated means (repeated measures ANOVA) of health, well-being (scale 1–7), and work satisfaction (scale 1–5) at baseline, 3 months, and 9 months after the implementation of activity-based workplaces (*n* = 246).

Self-assessed general health and well-being at work showed significant effects of time (*p* < 0.001); at the 3 months follow-up, health had increased, and well-being had decreased. Physical and psychosocial work satisfaction decreased significantly 9 months after the relocation compared with baseline (RM-ANOVA, *p* < 0.01, see Table 2 and Figure 3).

### 3.3. Perceptions of Facilitating Factors and Barriers Related to SOC during Implementation

Factors that were perceived as facilitative or barriers for an ABW implementation were, for example, the degree of the sense of participation, support, and level of worker involvement in the implementation process and communication received. In the interviews, facilitating factors and barriers indicated a relation to the sense of coherence in different ways.

To facilitate meaningfulness and to control the perceived risk concerning concentration, a forum at the workplace where one could raise questions regarding the new office was proposed. Before the relocation, there was a perceived barrier of not being able to concentrate at work, but after the relocation, this was verified by some and dismissed by others, indicating that personal performance matters and individual differences were perceived as crucial to take into account in order to be manageable.

During the interviews, it was indicated that this forum for support was facilitating manageability, partly to understand the process, but also a forum to communicate concerns and special needs in the workplace. The different work zones make it possible to choose adjusted workstations. Changes in the employee collaborations in the ABW were addressed by the respondents in interviews before and after the transition. Aspects such as not knowing where colleagues were situated were addressed, but this also had a positive consequence of meeting and interacting with colleagues from other departments.

Employees participating in preparatory activities, such as workshops, also expressed an improved comprehensibility regarding the relocation and the process. All group interviews mentioned communication as contributing to their involvement in the implementation process. When someone directly receives communication or information, it was a facilitating factor, whereas passive communication, i.e., information on homepages, was a barrier among those who expressed a negative attitude towards the relocation. Some employees wanted the communication to be delivered to them and thought the communication initiatives regarding the relocation and activity-based work approach should come from someone other than themselves. See Table 3 for categories and excerpts. 

### 3.4. Effect of Indicators for SOC (Meaningfulness, Manageability, Comprehensibility) and Associations with Change in Health, Well-Being and Work Satisfaction after Relocation to an ABW 

**Figure 4:**
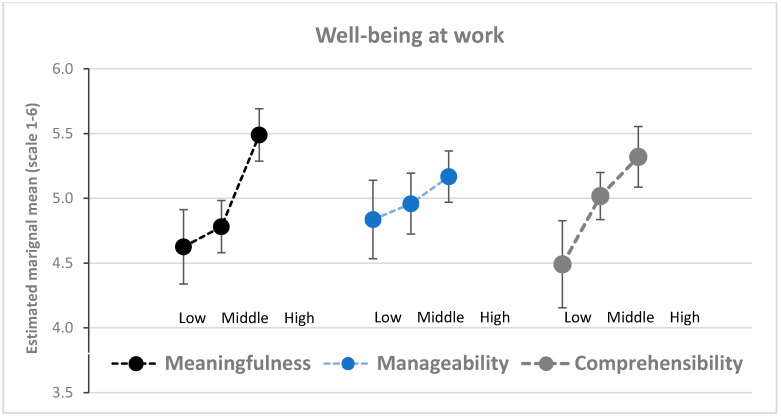
Estimated means of well-being in the workplace in groups (tertiles) with low, medium, and high meaningfulness, manageability, and comprehensibility (*n* = 246). Error bars indicate 95% confidence intervals.

A significant main effect was found in meaningfulness on perceived health (RM-ANOVA *p* < 0.001, see Table 4), showing a better health on average among those with high levels of perceived meaningfulness in the process. Main effects were also found with regard to meaningfulness and comprehensibility on the level of well-being in the workplace (RM-ANOVA *p* < 0.001, see Table 4), showing higher average levels of well-being in the workplace among those with high levels of perceived meaningfulness and comprehensibility in the process (Figure 4). 

Figure 4 illustrates how meaningfulness, manageability and comprehensibility are associated with well-being in the workplace. The values for well-being in the workplace are higher in tertiles with high indicators for SOC.

Meaningfulness and comprehensibility showed significant main effects on satisfaction with the physical and psychosocial work environment, with higher satisfaction rates among those experiencing high meaningfulness/comprehensibility (Table 5). 

**Figure 5:**
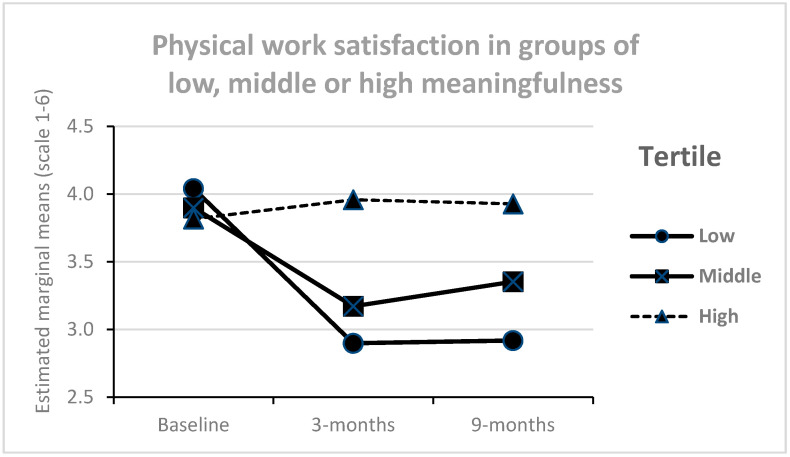
Physical work satisfaction over time in groups with different levels of perceived meaningfulness in the process (*n* = 246).

**Figure 6:**
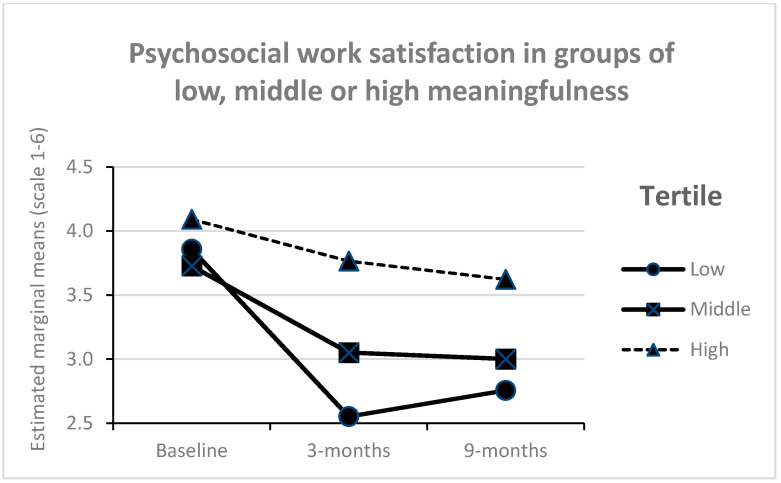
Psychosocial work satisfaction over time in groups with different levels of perceived meaningfulness in the process (*n* = 246).

Meaningfulness interacted with time (Figure 5), showing that the reduction in physical work satisfaction over time was smaller among those with high levels of perceived meaningfulness compared to middle and low levels (F = 12.0, *p* < 0.001, partial eta square 0.05). A similar interaction was found on psychosocial work satisfaction (Figure 6), showing that reductions in psychosocial work satisfaction over time was smaller among those with high levels of perceived meaningfulness compared to middle and low levels (F = 9.5, *p* 0.002, partial eta square = 0.04). No other interaction effects were significant (*p* > 0.05).

## 4. Discussion

This is, to the best of our knowledge, the first study addressing the indicators for SOC, meaningfulness, manageability, and comprehensibility, in relation to health, well-being and satisfaction during a relocation to an ABW. Regarding the implementation of ABWs, we found that indicators for SOC significantly increased 9 months after the relocation to an ABW was completed compared to before. Work satisfaction decreased, while health and well-being were unchanged. On average, a higher SOC was associated with better health, well-being, and satisfaction during relocation. Higher perceived levels of meaningfulness mitigated the negative effects of relocation on work satisfaction.

Regarding changes in the indicators for SOC during relocation, we found increases in the perceived manageability, comprehensibility, and meaningfulness 3 and 9 months after relocation. This means that perceptions regarding relocation seem to change over time, being higher after relocation than prior to it taking place. This indicates that the SOC for relocation changes during the relocation process and suggests that employees begin to understand what it is like to work in ABWs. From the focus group interviews, we found that the activities prior to the intervention, implemented with the goal of preparing employees for the relocation, contributed to the perceived meaningfulness, provided they participated. On the other hand, some expressed that they did not believe in ABWs, which contributed to the forming of a barrier in terms of involvement in the process. 

Comprehensibility might be facilitated through information and a sense of understanding as to why and how ABWs are beneficial. According to the respondents, manageability might be created through tools: for instance, office rules on how to take a new approach to work. This result adds to the knowledge produced by previous research, indicating that SOC can be used as a managerial tool [30]. However, we found no significant effects of the perceived manageability on health, well-being, and work satisfaction during relocation, which may be due to a sense of already (baseline) having disposable resources for working in an activity-based fashion such as mobile phones and digital files instead of paper folders. 

The intention of the intervention was not explicitly to improve health, although we found a marginal change in health (increase) and well-being (decrease) at the 3 month follow-up compared to baseline, while no change was found at 9 months. This indicates that an ABW does not immediately have a marked impact on wellness. This is in line with previous research where one study found that office concepts have no or limited effects on health [14]. The follow-up period at 9 months may be too short to capture the changes in health and well-being. However, other studies suggest that employees adapt to the ABW over time [31], and thus we do not expect any further declines in health or well-being.

Physical and psychosocial work satisfaction also decreased. One explanation might be found in previous research, where one study found that people do not use equipment and work spaces as intended [20]. Relocation to ABWs and satisfaction has been studied previously [3], but for future studies, it would be interesting to explore what actually contributes to the changes in work satisfaction during relocation. 

Self-assessed health and well-being were better among those who perceived high levels of meaningfulness in the process of relocating. This suggests that a person who found the relocation to be meaningful had a better well-being throughout the intervention. However, it is also a possibility of reverse causation, i.e., that those with more wellness might perceive the process of relocating as more meaningful. The reduction in physical and psychosocial work satisfaction over time was also smaller among those with high levels of perceived meaningfulness prior to relocation. This decline in work satisfaction might be due to how meaningful one perceives the relocation to be, and that employees who perceive a process of change in the workplace as meaningful are more likely to experience better health than those who experience less meaningfulness. This result indicates that the perceived meaningfulness concerning relocation would act as a buffer against reduced work satisfaction after relocation to an ABW. Therefore, preparation activities should address factors that contribute to the understanding of why a change is made, tools to handle the change and thereby promote a feeling of meaning regarding the change among employees.

This study adds to existing knowledge [12,13,14,15,18,20] regarding implementations of ABWs, as we demonstrated the associations between the indicators for SOC and work satisfaction, well-being and to some extent for health. Additionally, SOC changes over time, becoming higher after the relocation than before. Thus, we suggest that meaningfulness, manageability, and comprehensibility are of importance for employees during the implementation process. 

### 4.1. Practical Implications

Since many interventions in the workplace are preceded by a planning phase and preparation, one recommendation is to plan activities that encourage perceived meaningfulness among employees in order to maintain or improve health and work satisfaction. Previous studies have demonstrated the importance of management being more active when implementing ABWs [32] and that management can contribute to creating a sense of meaningfulness.

In future interventions we recommend implementing preparation activities to facilitate the process, and that the activities should be regarded as mandatory. The preparation activities were not mandatory in the intervention in question, but based on the results, it should be considered as to whether some activities ought to be mandatory. For example, we found that those who did not participate in activities thought that a lack of information and involvement was a barrier, and that receiving information was a facilitating factor for comprehensibility. 

### 4.2. Method Discussion 

Using both quantitative and qualitative data is beneficial in this kind of study, where the qualitative data could contribute to a deeper understanding of the quantitative result. For instance, the manageability of the relocation increased when comparing the periods before and after the relocation. Explanations for this could be found in the interviews where employees described positive side-effects of the relocation, such as having stimulated meetings and interacting with colleagues from different departments. The effects on team collaborations in ABWs have also been shown in previous studies, e.g., [33].

For future studies, one could consider if using Antonovsky’s [5] sense of coherence scale [8] would be more appropriate than to construct one’s own adjusted questions. For this study, we believe that for data collection, it was better to reduce the number of questions in the questionnaire, particularly in order to relate the question to the specific workplace intervention. Another reason not to use Antonovsky’s sense of coherence scale in order to assess perceptions was that it especially addresses mental health [8]. In this study, questions were adapted to assess meaningfulness, comprehensibility, and manageability in relation to the process of relocating to an ABW.

The third data collection was performed 9 months after the relocation. A longer follow-up period would have given us more information about the long-term effects of the intervention.

Finally, the lack of a control group (e.g., an office that does not relocate to an ABW) is a limitation precluding inferences being made about the intervention effect. However, the repeated measures design is sufficient to study changes over time and the predictors for this change, which was the focus of this study.

### 4.3. Future Research

We recommend future studies on ABWs be conducted to further address indicators for SOC. SOC factors perceived as facilitative and as barriers for ABW implementation included the degree of sense of participation, support and level of worker involvement in the implementation process, communication received and meaningfulness. 

With regard to future research, it would be interesting to conduct a study on whether preparation activities before relocation correlate to SOC. Another lesson learned is that the research team should be involved as early as possible in workplace interventions in order to investigate and measure the success of the implementation of ABWs.

In this study, we did not take into account individual background variables or positions within the company. Previous research has studied whether gender and age impact the perception of workplace types [31]. There are some indications that this is the case, but we suggest that future research should explore if and how background variables impact the implementation and attitudes towards ABWs. 

## 5. Conclusions

To our knowledge, this is the first study exploring the associations between the indicators for sense of coherence and change in health, well-being and satisfaction during relocation to activity-based workplaces. Meaningfulness, manageability, and comprehensibility, regarding the implementation of ABWs, significantly increased when comparing the period before the relocation to 9 months after it had taken place. A higher meaningfulness mitigated the negative effects the relocation had on work satisfaction. 

Health and well-being were unchanged when compared to their levels before and after the relocation to an ABW. We found that physical and psychosocial work satisfaction decreased. 

On average, a higher level of perceived meaningfulness was associated with higher perceived health, well-being and physical/psychosocial work satisfaction during relocation. A higher comprehensibility also showed the associations with a better well-being and work satisfaction.

Facilitating factors and barriers during the relocation from traditional offices to an ABW included support, tools on how to work in an ABW environment, communication and appropriate preparatory activities ahead of the relocation.

We conclude that employees who perceive a changing process in the workplace as meaningful are more likely to experience better health, well-being and work satisfaction than those who feel less meaningfulness regarding ABWs. Therefore, our recommendation is to ensure that meaningfulness and comprehensibility regarding change are high when launching an intervention. Meaningfulness might be promoted by preparatory activities, such as workshops and seminars, which provide meaning and understanding for the change.

## Figures and Tables

**Table 1:** Repeated measure ANOVA comparing meaningfulness, manageability and comprehensibility between baseline, 3 months, and 9 months after relocation to activity-based workplaces (*n* = 246).

Tests of Within-Subjects Effects, Repeated Measure ANOVA			
	**F**	***p***	$\eta_{p}^{2}$
Meaningfulness	12.2	<0.001	0.05
Manageability	108.6	<0.001	0.31
Comprehensibility	29.0	<0.001	0.11

**Table 2:** Repeated measure ANOVA comparing health, well-being, and work satisfaction between baseline, 3 months, and 9 months after relocation (*n* = 246).

Tests of Within-Subjects Effects, Repeated Measure ANOVA			
	**F**	***p***	$\eta_{p}^{2}$
Health	8.4	<0.001	0.03
Well-being in the workplace	10.4	<0.001	0.04
Physical satisfaction	18.4	<0.001	0.07
Psychosocial satisfaction	36.0	<0.001	0.23

**Table 3:** Overview of categories and subcategories in the qualitative analysis supported by excerpts.

Categories	Subcategories	Excerpts	
		Facilitating Factors	Barriers
Meaning- Fullness	(I)Implementation activities contribute to the perception of meaningfulness (II) Don’t believe in new way of working	(I) “… I also think it is meaningful, definitely, and all the training and workshops, lectures that are both happening now and that will happen later, will be, I hope, meaningful at least” (Working group before relocation)	(II) I do not think it will be that much better [relocation], I hope so, I really hope that I will find things for my own part and for the group, so it will work great but I find it very hard to believe. (Manager after relocation)
Manage-ability	(I) Differences need to be taken into account (II) Support is needed (III) Toolbox for how to work activity based	(II) “… we have addressed a few times at workplace meetings whether there are any questions about it [relocation] and so on, and then I have also asked each person in employee interviews how they feel about this and if they have any concerns, something special or anything like that”. (Manager after relocation)	(I) “So, no consideration was given to an introvert or a person who wants safety, who wants calm and less interactions… I think about such people, how does such a person come into his own in this new environment” (Employees before relocation) (III) “…what tools are there, how do you work, what are the employees going to miss, get tips and advice, so we held several workshops discussed with managers who are used to working that way, what they had experienced. That way you, get the toolbox. I don’t think a lecture is enough. (Working group before relocation)
Compre-hensibility	(I) Received communication (II) Understanding why and how	(I) “We have received a lot of information, I think it is important because if I had moved into a new office with a new way of working without knowing how to behave or any expectations for how others should behave etc. then it would not have felt good. Nevertheless, I have now received information in many different stages… [description of the activities during implementation] so much information and preparation I think has been huge” (Employee after relocation) (II) “But it is positive that employers care and prepare for this [relocation] in many ways, as well as to get as many people as possible to be in the know. At the same time, it might be a little much in my opinion, but you can always just say no” (Employee before relocation)	(II)“… important to explain why we should make this change and why we have made this decision, its benefits and so on” (Employee before relocation)

**Table 4:** Effects of the indicators for sense of coherence (SOC) on perceived health and well-being in the workplace (*n* = 246).

	Health			Well-Being in the Workplace		
Main effects	F	*p*	$\eta_{p}^{2}$	F	*p*	$\eta_{p}^{2}$
Meaningfulness	12.3	<0.001	0.09	16.8	<0.001	0.12
Manageability	2.3	0.105	0.02	1.9	0.151	0.02
Comprehensibility	0.7	0.486	0.01	8.0	<0.001	0.06

Note: Repeated measure ANOVA: SOC × time baseline, 3 months, and 9 months.

**Table 5:** Effects of the indicators for sense of coherence (SOC) on perceived physical and psychosocial work satisfaction (*n* = 246).

	Physical Satisfaction			Psychosocial Satisfaction		
Main effects	F	*p*	$\eta_{p}^{2}$	F	*p*	$\eta_{p}^{2}$
Meaningfulness	18.6	<0.001	0.13	22.6	<0.001	0.16
Manageability	1.4	0.246	0.01	4.6	0.010	0.04
Comprehensibility	7.2	<0.001	0.06	13.9	<0.001	0.10

Note: Repeated measure ANOVA: SOC × Time baseline, 3 months, and 9 months.
